# Assessing Dietary Patterns, Lifestyle Practices, and Forest Foods with Bioactive Potential to Address Micronutrient Deficiencies and Noncommunicable Diseases in Northeast India

**DOI:** 10.3390/nu17203311

**Published:** 2025-10-21

**Authors:** Devaprasanna Patrick, Jancirani Ramaswamy, Thangavel Palanisamy, Raghu Raman, Prema Nedungadi

**Affiliations:** 1 Department of Food Science and Nutrition, Amrita School of Physical Sciences, Amrita Vishwa Vidyapeetham, Coimbatore 641112, India; devaprasannap@am.amrita.edu (D.P.);; 2 Department of Mathematics, Amrita School of Physical Sciences, Amrita Vishwa Vidyapeetham, Coimbatore 641112, India; 3 Amrita School of Business, Amrita Vishwa Vidyapeetham, Amritapuri 690525, Kerala, India; 4 Amrita School of Computing, Amrita Vishwa Vidyapeetham, Amritapuri 690525, Kerala, India

**Keywords:** dietary diversity, forest foods, micronutrients, noncommunicable diseases, bioactive compounds

## Abstract

Background: Natural solutions, such as locally available food resources (LAFRs) and nontimber forest products (NTFPs), are recognized for their bioactive potential in addressing health challenges. Despite Mizoram’s rich biodiversity, the population faces increasing risks of noncommunicable diseases (NCDs) and micronutrient deficiencies (MNDs). Methods: This cross-sectional study assessed priority dietary preferences, food group consumption, dietary diversity score, and lifestyle practices, alongside a review of the nutraceutical potential of LAFRs and NTFPs. A three-day dietary recall was analyzed using *t*-tests at a 5% significance level against standards from the Indian Council of Medical Research (ICMR). One-way ANOVA was further employed to examine potential differences in food group consumption among occupational, gender, and age groups. Results: Results revealed strong cultural preferences for carbohydrate-rich breakfasts and meat-based dinners, with lunch often skipped or replaced by snacks. Over 85% of participants reported inadequate intake of milk, fruits, pulses, and nuts. Compared with older and high-income women, younger women exhibited the lowest intake of food groups and nutrient-dense foods. Occupation significantly influenced dietary patterns, with heavy workers consuming more cereals but fewer micronutrient-rich foods. A shift from traditional to modern dietary and lifestyle practices was observed, influencing overall diet quality and long-term health outcomes. The mean Dietary Diversity Score (0–10) was 5.6 ± 1.3, indicating significant gender differences in diet variety (males: 5.8 ± 1.2; females: 5.4 ± 1.4; *p* = 0.04). The review highlights that LAFRs and NTFPs serve as valuable sources of antioxidants, anti-inflammatory compounds, and bioactives with antidiabetic and anticancer properties while also providing essential micronutrients. Conclusions: The findings reveal a marked dietary transition in Mizoram and underscore the urgent need for food-based strategies to address nutrient gaps and the growing burden of NCDs.

## 1. Introduction

The restoration and utilization of locally available food resources (LAFRs) and nontimber forest products (NTFPs) have gained attention for their therapeutic potential and contributions to sustainable livelihoods [1,2]. NTFPs are naturally occurring forest resources that are typically uncultivated, while LAFRs refer to food plants found within and around community living areas. LAFRs are highly nutritious, easily accessible, and often available at no cost, and they play a vital role in ensuring food and nutritional security [3]. Together, they represent sustainable sources of nourishment and health-promoting compounds deeply embedded in the traditional food systems of local communities.

Northeast India, globally recognized for its biodiversity, is a reservoir of indigenous knowledge systems encompassing food, agriculture, medicine, and natural resources. Mizoram is home to seven major tribes, including Hmar, Paihte, Pawi, Ralte, Lai, Mara, and Lusei, that constitute 94% of the state’s population. These communities maintain distinct food habits, culinary traditions, and cultural practices. They rely heavily on wild edible and medicinal plants for sustenance and health [4].

Despite this rich food culture and biodiversity, Mizoram faces a dual burden of noncommunicable diseases (NCDs) and micronutrient deficiencies (MNDs). The World Health Organization (WHO, 2021) has identified Mizoram as “the cancer capital of India,” with one in four males and one in five females at risk of developing cancer between the ages of 0 and 74 [5,6]. The ICMR—India Diabetes (ICMR-INDIAB, 2023) study further reports high incidence of hypertension (≥30%), obesity (≥25%), and adverse lipid profiles [7,8]. Concurrently, hidden hunger, a form of malnutrition arising from insufficient vitamins and minerals despite adequate caloric intake, remains a critical concern [9,10].

The Comprehensive National Nutrition Survey (CNNS, 2016–2018) documented deficiencies in vitamin A (47% of school-aged children), zinc, iodine, folate, and B12 in adolescents, along with vitamin D deficiency in 14–18% of children, 24% of adolescents, and 62.7% of elderly individuals [11,12].

Vitamin D status, in particular, may be modulated by genetic factors that influence individual responses to supplementation. These genetic variations should be considered when designing population-level nutrition programs aimed at correcting deficiencies [13]. Similarly, the National Family Health Survey (NHFS, 2019–2021) reported anemia prevalence exceeding 35% among women aged 30–39 years and affecting 46% of children under five years [13,14,15]. These micronutrient gaps highlight the urgent need for targeted policies and interventions.

Rapid dietary shifts driven by urbanization, increased consumption of processed food, and higher tobacco and alcohol use have further elevated health risks. Traditional foods such as saum (fermented pork fat), smoked meats, salted fish, and the use of baking soda in cooking have been linked to stomach carcinoma, type 2 diabetes and nutritional imbalances [5,16,17,18,19]. Conversely, evidence suggests that diets emphasizing nutrient-rich and functional foods improve nutrient adequacy. Leveraging both LAFRs and NTFPs, alongside community-based nutrition education, offers a promising approach to reducing these nutritional gaps [20].

Given the heavy reliance of local populations on forest foods and the region’s relatively low health indicators, this study employs a two-phase approach to examine the relationship between local resources and community-based nutritional solutions. The first phase involves a dietary survey assessing dietary preferences, food group consumption, dietary diversity score (DDS), and lifestyle practices among the local population. DDS quantifies the number of different food groups consumed over a reference period. It is a validated indicator of micronutrient adequacy and overall diet quality. Incorporating DDS into dietary analysis enables a comprehensive assessment of nutritional gaps in this population [21].

The second phase consists of a narrative literature review that explores the nutritional and nutraceutical potential of NTFPs and LAFRs. By integrating the survey results with evidence from the literature on the bioactive properties of these local foods, this study develops a cohesive framework linking dietary gaps to the health-promoting potential of LAFRs and NTFPs. This integration provides a culturally grounded and sustainable basis for designing interventions to address both micronutrient deficiencies and NCDs in Mizoram.

## 2. Materials and Methods

### 2.1. Study Design

The study was conducted in two distinct phases. Phase one employed a cross-sectional, mixed-methods design to assess the top five priority dietary preferences, food group consumption, dietary diversity score, and lifestyle practices in the targeted population. Phase two comprised a narrative literature review exploring the bioactive and nutraceutical profiles of NTFPs and LAFRs, aiming to link these functional attributes to the dietary gaps identified in phase one.

### 2.2. Phase 1: Cross-Sectional Study

#### 2.2.1. Study Locale

The field-based study was conducted in the Aizawl West–I subdivision of Aizawl District, Mizoram, using a convenience sampling approach for site selection. This approach was chosen due to practical constraints such as limited time and accessibility. The study area included urban and peri-urban localities such as Ramrikawn, Tanhril, Vaivakawn, and Chanmari West, offering a representative setting of socio-economic and cultural diversity within Mizoram’s urban population. The selected region was targeted to capture a diverse range of socio-economic, cultural, dietary, and lifestyle practices typical of urban and peri-urban Mizo populations. The demographic and cultural heterogeneity of the area enhances the representativeness of the sample within this geographic locale.

**Figure 1:**
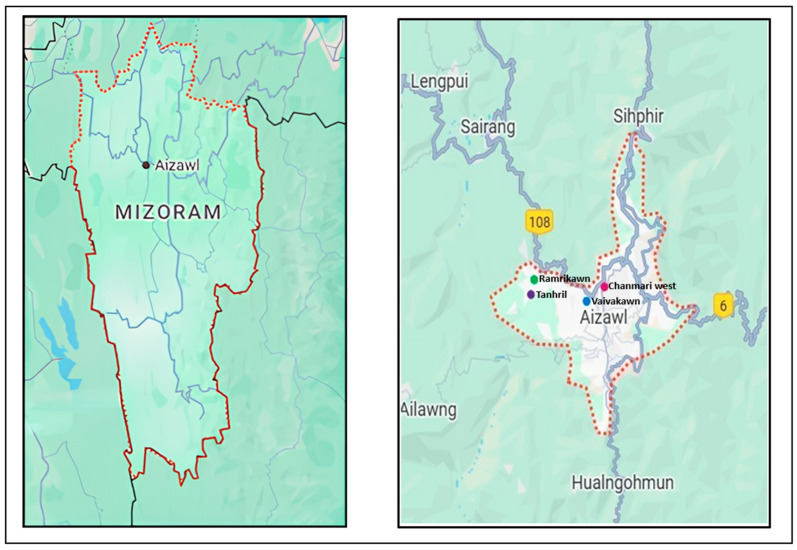
Map of the study area in the Aizawl West-I subdivision, Aizawl District, Mizoram.

Systematic random sampling was then applied to select households within this convenience-sampled area to reduce selection bias and improve internal validity. While findings are primarily applicable to the urban and peri-urban communities of Aizawl West-I, this approach provides meaningful insights into the dietary and lifestyle patterns of these local populations. The study location is illustrated in Figure 1.

#### 2.2.2. Participants and Sampling

A total of 170 household heads were selected through systematic random sampling, in which every third household was approached for participation. If the selected household did not meet the inclusion criteria, the next eligible household was considered before resuming the sampling interval. The final sample size of 170 was determined to balance methodological rigor and practical feasibility.

Although a priori power calculations are often used to justify sample size, this study adopted a systematic random approach within an accessible population of 647 eligible households, narrowed by inclusion criteria and feasibility over a six-month period. A similar study conducted in the Northeast region used 200 households to generate reliable community-level estimates without formal power analysis. Thus, the chosen sample size ensures adequate representation across diverse tribal groups while maintaining operational efficiency and internal validity [22].

Exclusion criteria included non-Mizo ethnicity, age below 26 or above 55 years, and lack of informed consent. Participants represented various tribal groups, including Hmar, Lusei, Ralte, Lai (Pawi), and Mara (Lakher) communities. Ethical approval for the study was obtained from the Mizoram University Ethics Committee (Approval No. MZU/HEC/2024/001).

#### 2.2.3. Data Collection and Analysis

Quantitative data were collected using a standardized three-day dietary recall questionnaire administered over consecutive days in the local languages by trained enumerators. Qualitative data collection involved focus group discussions to enhance understanding of food preferences and lifestyle habits. All survey instruments were piloted and refined for accuracy (see Appendix A).

Nutrient intake data from 170 participants were analyzed using Stats. Blue, comparing actual intake with the ICMR-Recommended Dietary Allowances (RDA) [23]. Statistical analyses included *t*-tests at the 5% significance level for mean comparisons. One-way ANOVA to examine differences in food group consumption among occupational and age groups. Analyses were conducted separately for males and females to identify nutritional imbalances. The Individual Dietary Diversity Score (IDDS) was computed based on ten food groups: cereals & millets; pulses; animal foods; green leafy vegetables; other vegetables; roots & tubers; fruits; milk & milk products; fats & oils; and nuts & seeds. For each participant, a food group received a score of 1 if consumed at least once during the three-day recall period (intake > 0 g) and 0 if not. DDS was calculated as the sum of these ten binary indicators (range 0–10). Participants were classified as having low (DDS ≤ 4), medium (DDS 5–6), or high (DDS ≥ 7) dietary diversity. DDS values were expressed as mean ± SD and compared between groups using an independent-samples *t*-test (with unequal variances where applicable). Categorical DDS classifications (low/medium/high) were compared using the χ^2^ test.

Lifestyle practices were assessed via a structured questionnaire adapted from the WHO STEPS Instrument, the Healthy Eating Index (HEI), and the Global Physical Activity Questionnaire (GPAQ), designed to capture risk factors associated with NCDs and MNDs.

### 2.3. Phase 2: Narrative Literature Review

A narrative review was conducted to compile existing peer-reviewed evidence on LAFRs and NTFPs and evaluate their potential in preventing or managing NCDs and MNDs. Scientific platforms such as Scopus, ResearchGate, ScienceDirect, etc., were searched using predefined keywords: “Forest Foods,” “Nutraceuticals,” “Functional Foods,” “Bioactive Compounds,” “Ethnomedicinal Plants,” “Dietary Patterns,” and “Northeast,” combined with Boolean operators “AND” and “OR.”

Peer-reviewed articles published between 2015 and 2024 were included. Publications outside this period or those not linking bioactive compounds to NCDs and MNDs were excluded. Of the 140 articles initially retrieved, 78 met the inclusion criteria and were reviewed for information on bioactive compounds, traditional preparation methods, plant parts used, and documented health benefits.

**Figure 2:**
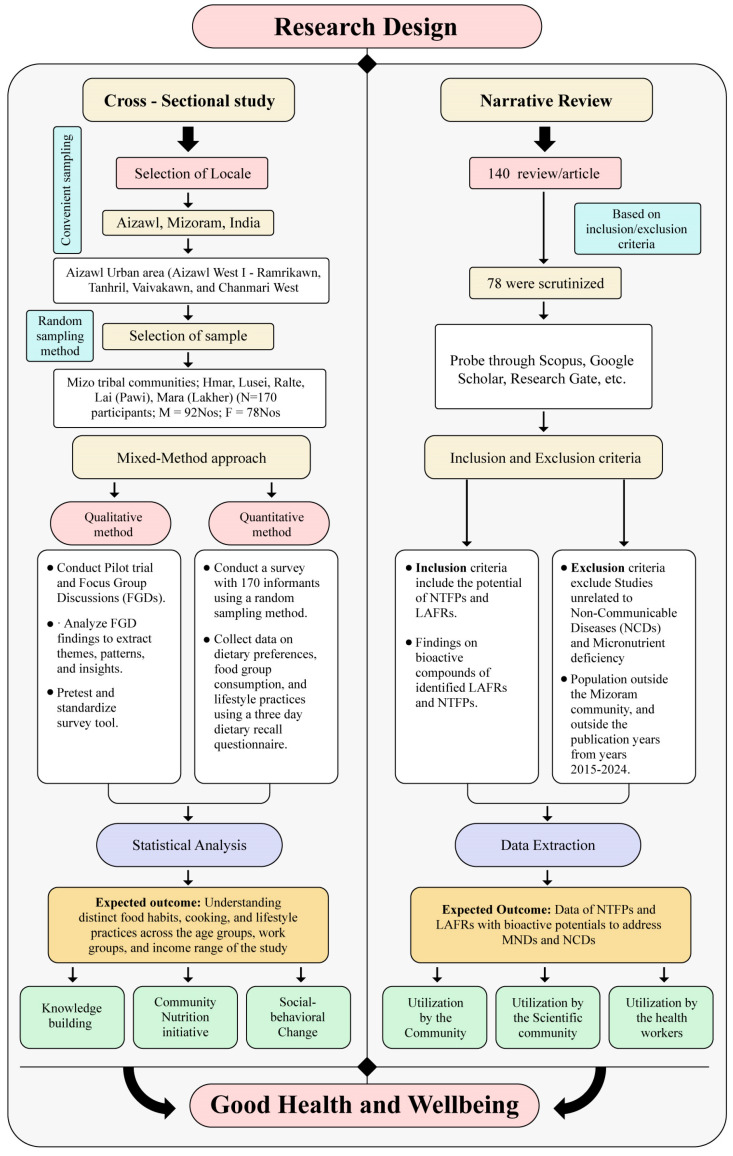
Research design framework—cross-sectional study and narrative review.

This phase aimed to directly connect the functional and bioactive properties reported in the literature with the nutrient gaps and NCD patterns identified in phase one. A schematic representation of the study design is provided in Figure 2.

## 3. Results and Discussion

The results from Phase 1 and Phase 2 were consolidated to provide an integrated understanding of the study objectives. Section 3.1, Section 3.2 and Section 3.3 present findings from Phase 1, which involved a dietary survey of 170 participants from tribal communities. These sections describe dietary preferences, food group consumption, DDS, and lifestyle practices among the study population. Section 3.4 summarizes Phase 2, the narrative literature review assessing the bioactive potential of local and forest foods in addressing nutrient gaps and NCDs identified in Phase 1.

Phase 1 included 170 participants (92 men and 78 women) aged 27–54 years, representing the productive (ability to work) age group. Participants were engaged in diverse occupations, including teaching, healthcare, driving, skilled labor, and domestic work, with monthly family incomes ranging from ₹7000 to ₹90,000 (Indian Rupees, INRs). This reflects the socio-economic heterogeneity of the studied population. Notably, 24% of household heads aged 35 years and above reported one or more diagnosed NCDs, underscoring the growing burden of chronic diseases. Despite the prevalence of NCDs, awareness of micronutrient deficiencies was limited, with only 8.12% of female participants recognizing conditions such as anemia.

### 3.1. Impact on Dietary Preferences

The dietary preferences of participants revealed strong cultural influences, particularly in breakfast and dinner choices, as represented in Table 1. Breakfasts were dominated by carbohydrate-rich items, while dinners commonly included soups, stews, or meat-based dishes. Lunch was often skipped or replaced with processed snacks and tea. Such patterns, combined with convenience-oriented food choices, reflect an overreliance on high-fat and high-sugar foods, contributing to micronutrient deficiencies and the early onset of NCDs, such as diabetes and cardiovascular diseases (CVDs) [5]. These behavioral shifts align with broader nutritional transitions observed among tribal and urbanizing populations in Northeast India.

### 3.2. Impact on Food Group Consumption Among Mizo Tribal Communities

**Figure 3:**
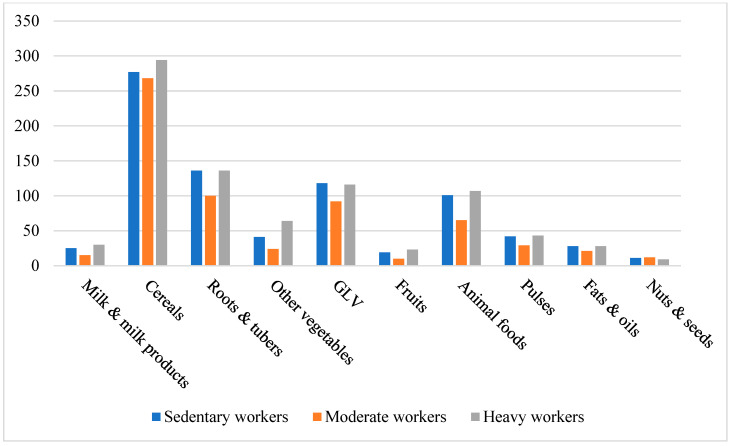
Food Group Consumption vs. Workgroups of Males.

**Figure 4:**
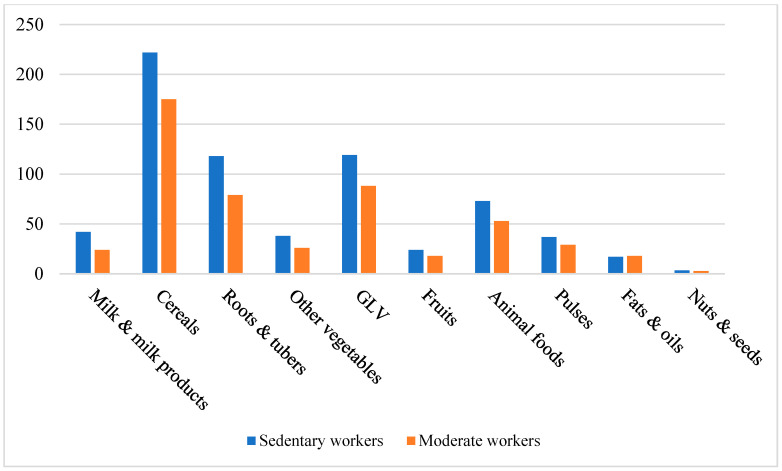
Food Group Consumption vs. Workgroups of Females.

**Figure 5:**
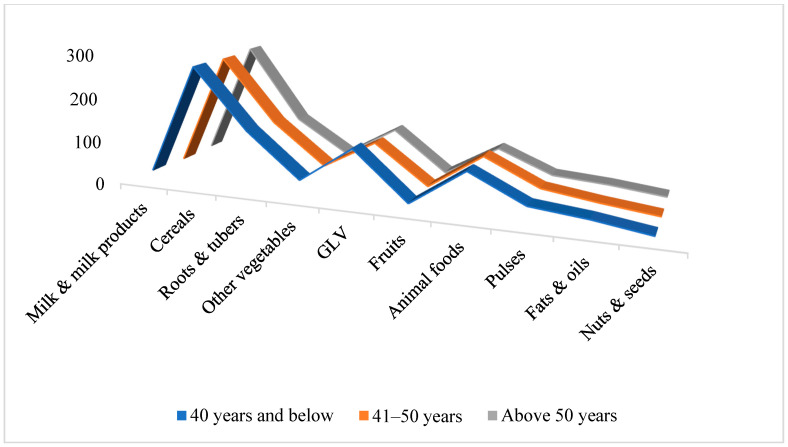
Food Group Consumption vs. Age Groups of Males.

**Figure 6:**
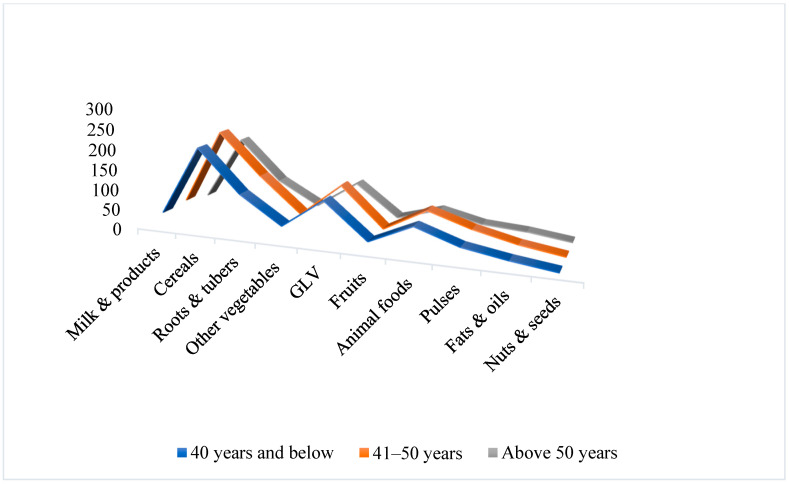
Food Group Consumption vs. Age Groups of Females.

Comparison with ICMR-RDA standards revealed marked dietary imbalances. Participants consistently overconsumed cereals, animal-source foods, and fats/oils, while intake of fruits, dairy, pulses, nuts, roots, and other vegetables was critically inadequate. Green leafy vegetables were the only food group consumed in adequate amounts. Income influenced female dietary quality more than male, with higher-income women showing marginally improved consumption of dairy, fruit, and animal products. These patterns are detailed in Table 2 and illustrated in Figure 3, Figure 4, Figure 5 and Figure 6. Such dietary deficits are linked to weakened immunity and heightened susceptibility to chronic diseases, including cardiovascular diseases, diabetes, and metabolic disorders, as documented in recent regional and global dietary studies [24,25,26].

#### Dietary Diversity Score Assessment

Individual Dietary Diversity Scores (DDS) were calculated for each participant using ten ICMR-defined food groups based on the three-day dietary recall to evaluate overall diet quality and micronutrient adequacy. The mean DDS was 5.6 ± 1.3, and it is represented in Table 3. Males exhibited significantly higher DDS values than females (5.8 ± 1.2 vs. 5.4 ± 1.4). The *p*-value for the independent-samples *t*-test comparing DDS between males and females is 0.04, indicating gender disparities in dietary variety. Low diversity (DDS ≤ 4) was observed among 22.4% of participants, medium diversity (DDS 5–6) among 45.8%, and high diversity (DDS ≥ 7) among 31.8%.

These results reinforce quantitative findings of limited consumption of pulses, fruits, milk, and nuts & seeds, which contributed to low to moderate DDS across the majority. An overdependence on cereals and animal-source foods was evident. The high percentage of participants with low DDS highlights widespread inadequate dietary variety, elevating the risk of micronutrient deficiency and NCDs. Such gender differences underscore the need for targeted nutrition interventions.

Further analysis revealed consistent nutrient gaps across workgroups. Moderate workers consumed particularly low quantities of milk, fruits, roots, tubers, and pulses. None of the groups met the ICMR recommendations for these food categories. Cereal intake was slightly higher among heavy workers, while green leafy vegetable intake was the highest among sedentary workers. Figure 3 and Figure 4 illustrate these variations. One-way ANOVA (males) and *t*-tests (females) revealed significant differences across workgroups. The *p*-values obtained from these analyses are presented in Table 4.

Significant age-related differences were observed in the intake of milk, vegetables, pulses, and animal foods, with older women showing greater adherence to ICMR recommendations. These observations are illustrated in Figure 5 and Figure 6. The population exhibits consistently high cereal and animal food group consumption coupled with low fruit and vegetable intake, revealing a double burden of micronutrient deficiency and increased NCD risks. Adequate intake of multiple micronutrients is crucial to support immune function and reduce infection risk throughout life. Pulses like beans and lentils, rich in resistant starch and fiber, offer prebiotic benefits that promote gut and metabolic health, especially in aging populations [27,28]. One-way ANOVA results for male and female age groups are summarized in Table 5.

Income also influenced dietary diversity. High-income residents had better access to nutrient-rich foods such as milk and meat, highlighting income as a determinant of diet quality [29,30]. Among males, food habits remained relatively stable across income and age groups. Although higher income was associated with greater diet variety among females, ANOVA showed no significant differences in nutrient intake. Limited awareness of nutrient deficiencies among participants further contributes to inadequate consumption, emphasizing the need for nutrition education.

### 3.3. Impact on the Prevailing Lifestyle Practices of the Mizo Tribes

Lifestyle practices among the Mizo tribes revealed substantial behavioral changes impacting health outcomes. Table 6 contrasts favorable practices such as consuming local leafy vegetables, traditional preservation techniques, and home gardening with unfavorable behaviors including widespread consumption of fermented pork fat (sa-um), processed foods, and commercial baking soda. The use of baking soda in soup (bai) preparations may reduce mineral absorption, worsening calcium, iron, and zinc deficiencies and increasing risks of hypertension, CVD and diabetes. Low dairy consumption contributes to calcium and vitamin D insufficiency, heightening the risk of osteoporosis. Sedentary lifestyles, substance use, and stress, especially among younger adults, are associated with higher incidences of obesity, metabolic syndrome, and mental health issues [5,16,19,31,32].

Additionally, the community’s limited awareness of nutrition and health impacts compounds the problem. These dietary and lifestyle transitions have also reduced exposure to natural bioactive compounds abundant in regional NTFPs and LAFRs [5,33]. These findings support the exploration of local and forest foods as preventive nutritional resources.

### 3.4. Bioactive Potential of NTFPs and LAFRS

To address the observed nutrient gaps and lifestyle-related health challenges, the study examined the nutritional and nutraceutical potential of regional NTFPs and LAFRs. The dietary survey revealed critical shortfalls in essential nutrients such as iron, calcium, and vitamins D, A, and C, which are strongly associated with anemia prevalence. NTFPs such as *Oroxylum indicum*, *Centella asiatica*, and *Zanthoxylum asiaticum* possess potent antioxidant, anti-inflammatory, and metabolic regulatory properties that may help mitigate the NCD risks, including diabetes and sickle-cell anemia. Similarly, LAFRs, such as Beta vulgaris, contribute to improved hemoglobin levels, enhanced immune function, and reduced micronutrient deficiencies [34,35,36,37,38]. Beyond nutrient adequacy, these NTFPs and LAFRs contain bioactive compounds with potential roles in mitigating chronic diseases such as diabetes and cancers, adding further justification for their integration into dietary interventions.

The functional profiles of NTFPs and LAFRs presented in Table 7 and Table 8 demonstrate their capacity to diversify diets and enhance nutrient intake and reduce NCD risk. Their documented therapeutic activities justify their integration into preventive health strategies and community nutrition programs. Despite their availability, awareness and utilization remain low, with more than one-fifth of participants reporting low dietary diversity. Similar findings among vulnerable tribal communities reinforce the importance of leveraging traditional ecological knowledge and access to nutrient-rich indigenous foods as sustainable, food-centered interventions. This alignment between nutrient intake findings and the functional attributes of local bioactive foods provides a strong evidence base for sustainable interventions in Mizoram [33,39,40,41].

## 4. Challenges and Gaps

This study employed convenience sampling for site selection because of practical considerations such as limited time, budget, and accessibility. Although this method allowed efficient data collection from urban and peri-urban populations and helped capture diverse socio-economic and cultural groups, convenience sampling inherently limits the representativeness of the sample and the ability to generalize findings to broader populations. To mitigate this, systematic random sampling was applied within the convenience-sampled area, improving the internal validity by structuring household selection and reducing selection bias. Despite these concerns, participant reports and observed dietary patterns closely align with regional dietary behaviors reported in comparable urban contexts. This provides reassurance regarding the relevance and applicability of the findings within similar socio-cultural environments. The current study offers valuable insights into dietary diversity and lifestyle practices that may inform future research employing more rigorous sampling methods across broader populations.

Furthermore, the nutritional potential of Local Available Food Resources (LAFRs) and Non-Timber Forest Products (NTFPs), validated biochemically, remains insufficiently explored. Biochemical analyses and biomarker-based validation are essential to thoroughly assess the nutraceutical attributes of local foods. Such integrated approaches will strengthen the evidence base, deepen scientific understanding, and guide the design of culturally sensitive and context-specific nutritional interventions.

## 5. Conclusions

The findings reveal emerging nutritional vulnerabilities within the Mizo tribal population, largely driven by a shift from traditional diets toward highly processed, energy-dense foods that are high in salt, sugar, and fat. This dietary transition has intensified MNDs and contributed to the rising burden of NCDs. The coexistence of poor dietary diversity, declining reliance on traditional knowledge, and limited awareness of nutritional health underscores the need for immediate intervention.

Despite living in a biodiversity-rich environment, the population has not fully leveraged indigenous food resources to enhance dietary diversity. The observed low to moderate DDS illustrates a significant gap between the abundance of nutrient-dense resources and their limited consumption in daily diets. By linking the population’s nutrient intake data with scientific evidence on the bioactive compounds of NTFPs and LAFRs, this study identifies culturally relevant strategies to mitigate MNDs and NCD risk.

These results emphasize the urgent need to revitalize and integrate traditional nutrient-rich foods into modern dietary patterns. Multisectoral strategies are essential to bridge traditional food knowledge with modern nutrition through inclusive policy measures.

## 6. Policy Implications

The integration of indigenous, nutrient-rich foods into national and state-level public health programs such as the Integrated Child Development Services (ICDS), Mid-Day Meal schemes, POSHAN Abhiyaan, and Anemia Mukt Bharat are critical for improving the nutritional status of vulnerable populations. Prioritizing the inclusion of local food resources beyond staple cereals in these programs is essential to effectively address micronutrient deficiencies, which disproportionately affect women and moderate work groups.

In addition, formulating dietary guidelines tailored to local agroecological and cultural contexts is vital. To achieve this goal, collaboration among the Ministry of Health and Family Welfare, the Ministry of AYUSH, and research bodies such as ICMR-NIN can facilitate the inclusion of locally available forest resources (LAFRs) and nontimber forest products (NTFPs) in these guidelines.

Recent advances in personalized health monitoring underscore the value of integrating traditional dietary practices and bioactive indigenous foods into community health strategies, enabling more effective management of nutrition and lifestyle-related challenges in vulnerable populations [41].

In line with these efforts, empowering frontline health workers through focused training programs will support the promotion of traditional foods and foster sustainable dietary behaviors within communities. Furthermore, nutrition communication strategies must be gender-sensitive and utilize digital platforms to increase awareness of micronutrient deficiencies, particularly among women.

Addressing the erosion of traditional food knowledge is also critical. Community-based interventions should promote informed adaptations of cultural dietary practices. To sustain these practices, policy incentives must encourage sustainable harvesting methods that protect biodiversity and improve the livelihoods of indigenous populations. Integrating the cultivation and regulated use of nutrient-dense wild edibles into agroforestry and biodiversity conservation programs further ensures long-term ecological balance and resource availability.

To monitor progress and disparities, establishing localized, gender- and age-disaggregated nutrition and health surveillance systems will enable effective evaluation of interventions. This data-driven approach will support ongoing policy refinement.

Finally, strengthening the scientific foundation for these policies through interdisciplinary research on bioactive compounds of indigenous foods and their health benefits is imperative. Such research will underpin evidence-based nutraceutical interventions aligned with preventive healthcare goals. These initiatives align with the United Nations Sustainable Development Goals, particularly zero hunger, health and well-being, and sustainable use of terrestrial ecosystems.

Strategic funding and institutional collaboration remain pivotal in translating research findings into actionable policies and practices, ensuring that traditional food systems are harnessed to improve nutritional outcomes in culturally and environmentally sustainable ways.

## Figures and Tables

**Table 1:** Top 5 priority dietary preferences among the Mizo tribes (*n* = 170 adults). Notes: Colored circles indicate the most significant nutritional attributes of each food based on typical preparation and dominant ingredients. Only major attributes are shown to prevent visual overcrowding.

Priority Options	Breakfast/ Local Name	Lunch/ Local Name	Dinner/ Local Name	Snacks Local Name	Dominant Nutritional Attributes
1	Sawhchiar (Mizo fried rice)	Tea and bread	Rice and Bai (Green leafy vegetable soup)	Mizo sticky rice cakes	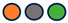
2	Chowmein (Stir-fried noodles)	Noodles	Rice and Bawngchawl (Mizo-style curry)	Pancakes	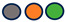
3	Rice and Vawksa rep (Smoked pork)	Tea and biscuits	Rice and Chamthong (Mizo veggie stew)	Panchu pui (Deep-fried snack made with rice and seasonings)	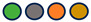
4	Rice, potato, and Dal	Tea and chips	Arsa Buhchiar (Rice cooked with chicken—porridge)	Nghapui (Fried/roasted peanuts seasoned with spices)	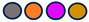
5	Bawmchawlini (Mizo-style rice with meat porridge)	Bread and Jam	Rice and sausage (pork)	Thums (Fermented bamboo shoots)	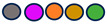

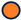
 Rich in CHO; 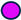 Rich in protein and healthy fats; 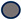 Highly refined carbs and added sugars; 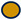 Unhealthy fat and high calories; 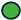 Rich in vitamins and minerals.

**Table 2:** Food group consumption of Mizo tribes for 3 consecutive days (*n* = 170 adults).

Food Groups	Male *n* = 92 Nos (27–54 Years)			Female *n* = 78 Nos (27–54 Years)		
	ICMR RDA (g/day)	Mean Actual Intake (g)	%Excess /Deficit	ICMR RDA (g/day)	Mean Actual Intake (g)	%Excess/ Deficit
Cereals	275	278 ± 45	+1.09	200	208 ± 62	+4
Pulses	80	40 ± 16	−50	60	34 ± 17	−43
Animal foods	80	96 ± 44	+20	60	67 ± 32	+11.66
Green leafy vegetables	100	114 ± 32	+14	100	109 ± 36	+9
Other Vegetables	200	130 ± 53	−35	200	105 ± 43	−47.5
Roots and tubers (excluding potato)	100	41 ± 23	−58.33	100	35 ± 13	−65
Fruits	150	18 ± 9	−88	150	22 ± 14	−85.33
Milk & milk products	300	24 ± 14	−92	300	36 ± 19	−88
Fats and oils	25	27 ± 11	+8	15	17 ± 4	+13.33
Nuts and seeds	30	11 ± 11	−63.33	30	3 ± 7	−97

Source: ICMR-NIN Expert Group on Nutrient Requirements for Indians, Recommended Dietary Allowances (RDAs) and Estimated Average Requirements (EAR)—2020. Notes: Millets were excluded from food group consumption because they are not commonly available in Mizoram.

**Table 3:** Distribution of Dietary Diversity Scores among males and females (*n* = 170). Notes: Values are presented as mean ± standard deviation, range, and frequency (%).

DDS Parameter	Males (*n* = 78)	Female (*n* = 92)	Total (*n* = 170)
Low diversity (≤4), *n* (%)	13 (16.7%)	25 (27.2%)	38 (22.4%)
Medium diversity (5–6), *n* (%)	42 (53.8%)	36 (39.1%)	78 (45.8%)
High diversity (≥7), *n* (%)	23 (29.5%)	31 (33.7%)	54 (31.8%)
Mean DDS ± SD	5.8 ± 1.2	5.4 ± 1.4	5.6 ± 1.3

**Table 4:** *p*-values from ANOVA (males) and *t*-tests (females) for food group consumption among workgroups.

Food Groups	*p*-Values from ANOVA (Males)	*p*-Values from *t*-Tests (Females)
Milk & milk products	0.0068	0
Cereals	0.4659	0.0156
Roots & tubers	0	0.0002
Other vegetables	0.0011	0.0001
Green leafy vegetables	0.0011	0.0003
Fruits	0.0005	0.0132
Animal foods	0.006	0.0009
Pulses	0.1826	0.0019
Fats & oils	0.0039	0.0936
Nuts & seeds	0.8495	0.0196

**Table 5:** *p*-values from ANOVA for food group consumption among males and females age groups.

Food Groups	*p*-Values (Males)	*p*-Values (Females)
Milk & milk products	0.218	0.0363
Cereals	0.9467	0.1018
Roots & tubers	0.7196	0.0856
Other vegetables	0.0854	0.0002
Green leafy vegetables	0.0262	0.1238
Fruits	0.2105	0.5822
Animal foods	0.265	0.0066
Pulses	0.1552	0.0009
Fats & oils	0.1403	0.347
Nuts & seeds	0.5449	0.1103

**Table 6:** Favorable and unfavorable prevailing lifestyle practices, recorded among the Mizo tribal community (*n* = 170 adults).

Category	Favorable Practices	Unfavorable Practices
**Eating Habits**	Inclusion of Local Foods	High Salt Intake
	Consumption of green leafy vegetables	Excessive Sugar Consumption
	Consumption of Bai (Boiled Vegetables)	Processed Foods
	Consumption of fermented foods	Skipping Meals
	Moderate use of spices	Unbalanced Snacking
	Preparation and usage of ash filtrate from the plant’s stems and leaves	Use of commercial baking soda
	Low oil consumption	Increased use of saum (fermented pork fat)
	Utilization of seasonal resources	Smoked meat consumption
	Intake of common vegetables	Less intake of milk, fruits, roots, and tubers
**Lifestyle** **Factors**	Utilization of traditional food preservation techniques	Sedentary Lifestyle
	Promotion of Home gardening	Alcohol and Substance Abuse
	Purchase of Local produce to support local farmers	Stress
	Living close to rich natural resources	Smoking
	Practice of sustainable resource management	Less physical activity
**Cooking Practices**	Utilization of fresh forest foods	Declining traditional cooking practices such as foraging of wild ingredients, traditional preservation methods, steaming of bamboo, etc.
	Boiling, steaming, grilling, and stewing methods	Emerging Reliance on Packaged Foods

Source: WHO STEPS instrument, Healthy Eating Index (HEI), Global Physical Activity Questionnaire (GPAQ).

**Table 7:** List of NTFPs, major bioactive compounds, and their potential usage. Note: Detailed bioactive compound subclasses and extended phytochemical profiles for each species are provided in Supplementary Table S1 to maintain clarity and focus in the main text.

S. No	Species Name; Local Name; Common Name	Parts Used/ Preparation & Consumption Methods	Addressing the Type of NCDs/Micronutrient Deficiency Specific to the Plant	Major Bioactive Compounds	References
**1.**	*Callicarpa arborea Roxb*; Beauty berry; Hnah kiah	Leaf, Bark/Decoction	Diabetes, Cancers	clerodane, diterpenoid, flavonoids, tannins	[42,43,44]
**2.**	*Catharanthus roseus* (L.); Kumtluang; Bright eyes	Leaf, whole plant, Root/Juice, decoction	Diabetes, Hypertension, CVDs, Cancers	vincristine, vinblastine, vindolidine	[42,45,46]
**3.**	*Clerodendrum glandulosum Lindl*; Phuihnam; Hill glory bower	Leaf, stem, root/Decoction	Hypertension, Diabetes, Obesity, CVDs	caffeic acid, luteolin, apigenin	[42,47]
**4.**	*Helianthus annus*; Sunflower; Ni-hawi	Seeds, stem, roots, leaf, flower/Raw, Oil, Meal	Cancer, obesity, diabetes, hypertension, CVDs/Bone health	apigenin, quercetin, caffeic acid	[42,48,49]
**5.**	*Oroxylum indicum*; Broken bones; Ar-chang-kawm	Bark/Decoction	CVDs, Cancer, Diabetes, Asthma	baicalein, oroxylin A, chrysin, scutellarin, ellagic acid	[42,50]
**6.**	*Centella asiatica*; Indian Pennywort; Darbengbur/lambak	Leaf, Stem/Decoction, Powder	Diabetes, Cancers	quercetin, madecassoside, kaempferol, asiaticoside, flavonoids	[42,51,52]
**7.**	*Artemisia vulgaris*; Mugwort; Sai	Plant/Decoction	Cancers, Diabetes, Hypertension, Obesity	artemisinin, apigenin, quercetin	[42,53,54]
**8.**	*Zanthoxylum asiaticum*; Orange climber; Ching-it	Stem, bark, leaves, roots/Decoction	Cancers/Sickle cell anemia	alkaloids, phenols, tannins, flavonoids	[42,55,56]
**9.**	*Mikania micrantha*; Bitter vine; Japan-hlo	Leaves, Whole plant/Decoction	Stroke, Hypertension, Diabetes, Cancers	saponin, phenols, flavonoids	[42,57,58]
**10.**	*Alstonia scholaris* (L.) *R*.; Devil’s tree; Thuamriat	Bark, stem, leaves, and roots/Decoction	Cancers, Diabetes, Heart disorders, Hypertension	alkaloid, flavonoids, triterpenoids	[42,59,60]
**11.**	*Cannabis sativa L.*; Hemp; Trip Kanza	Seeds	Cancers, CVDs, Diabetes, Hypertension/Bone health	cannabinoids, terpenoids, polyphenols	[42,61,62]
**12.**	*Magnolia champaca* L.; Champak; Ngiau	Stem bark, Leaf/Maceration	Hypertension, Cancers	flavonoids, phenols, alkaloids	[42,63,64]
**13.**	*Rubus ellipticus*; Yellow Himalayan raspberry; Zawngṭa	Leaves, fruits, shoots, root bark/Decoction, Juice	Cancer, Diabetes	quercetin, ellagic acid, catechin	[65,66]
**14.**	*Termitomyces heimii*; Wild mushroom; Pasawntlung	Edible portion/Cooked	Hyperlipidemia, Cancers	quercetin, saponins, tannins	[67,68]
**15.**	*Cinnamomum tamala*; Indian bay leaf; Theipui	Leaves	Hypercholesterolemia, Cancer, CVDs, Diabetes	terpenoids, flavonoids, phenolic acids	[69,70,71]

**Table 8:** Lists of LAFRs, major bioactive compounds, and their potential uses. Note: Detailed bioactive compound subclasses and extended phytochemical profiles for each species are provided in Supplementary Table S2 to maintain clarity and focus in the main text.

S. No	Species Name; Local Name; Common Name	Parts Used/ Preparation & Consumption Methods	Addressing the Type of NCDs/Micronutrient Deficiency Specific to the Plant	Major Bioactive Compounds	References
1.	*Beta vulgaris* L.; Beetroot; Beetroot	Rhizome/Raw, Juice	Cancers/Anemia	β-carotene, lutein, lycopene	[42,72,73]
2.	*Carica papaya* L.; Thingfanghma; Papaya	Seed, Fruit, Leaf, Sap/Paste, Raw	Cancers, Diabetes, Obesity, CVDs, and Asthma	quercetin, caffeic acid, lycopene	[72,74]
3.	*Citrus maxima*; Sertawk; Pomelo	Whole plant, whole fruit, albedo, Peel, Leaf, Pulp, and Seed/Raw	Hypertension, Cancer, Obesity/Osteoporosis	naringin, hesperidin, quercetin	[72,75,76,77,78]
4.	*Citrus limon* (L.); Nimbu; Lemon	Fruit, Stem, Leaf, Peel/Juice, Decoction	Neurodegenerative diseases, CVDs, Hypertension, diabetes, cancer/Scurvy, osteoporosis	ascorbic acid, polyphenols, flavonoids	[72,79,80]
5.	*Colocasia esculenta* (L.); Dawl; Elephant ear	Stem, shoots, flowers, sap, bulbs, leaves/juice, boiled, stewed	Cancer, Diabetes/Anemia	terpenoids, alkaloids, flavonoids	[42,72,81]
6.	*Crassocephalum crepidioides*; Buar thau; Fireweed ragleaf	Leaf/Paste	Diabetes, Cancer, Obesity, Hypertension/Anemia	gallic acids, catechin, rutin	[42,72,82,83,84]
7.	*Cucurbita maxima Duchesne*; Mai; Pumpkin	Fruit, flesh, seed, leaf, and peel/raw, boiled, steamed	Diabetes, Obesity, Cancers, Hypertension, CVDs/Anemia	gallic acid, kaempferol, quercetin	[35,42,72,85,86]
8.	*Curcuma longa* L.; Aieng; Turmeric	Rhizome/Powder, Juice	Cancers, Diabetes, CVDS	curcumin, ar-turmerone	[42,72,87]
9.	*Cucumis sativus* L.; Fanghma; Cucumber	Leaf, flesh, seeds, Skin/Raw, decoction	Hypertension, Diabetes, Cancers	cucurbitacin B	[42,72,88,89]
10.	*Dillenia pentagyna Roxb*; Kaihzawl; Elephant Apple	Leaf, Bark, Fruits/Decoction	Cancer, Diabetes, CVDs	lupeol, naringenin, β-sitosterol, gallic acid	[42,72,90]
11.	*Zingiber officinale*; Ginger; Sawhthing	Rhizome/Raw, Decoction	Diabetes, CVDs, Cancers/Anemia, Osteoporosis	gingerol, shogaol, zingerone, β-sitosterol, quercetin, rutin	[42,72,91,92]
12.	*Phyllanthus emblica* L.; Indian Gooseberry; Sunhlu	Fruit/Raw, Juice	Diabetes, Cancers/Anemia, Scurvy	ascorbic acid, ellagic acid, gallic acid	[42,72,93,94]
13.	*Hylocereus undatus*; Dragon fruit; Dragon fruit	Flesh, seeds, and peels	Obesity, Diabetes, Cancers, CVDs/Anemia	quercetin, ascorbic acid, β-carotene	[42,95,96,97]
14.	*Ananas comosus* L.; Pineapple; Lakhuih	Pulp, Leaf, Root, Skin, core, stem/Raw, Juice, Paste	CVDs, Diabetes, Cancers/Bone and Oral health	bromelain, vitamin C, phenols	[42,98,99,100]
15.	*Phyllostachys edulis*; Bamboo shoot; Mautuai	Edible portion/Boiled	Coronary heart disease, Cancers, Diabetes, obesity/Micronutrient deficiencies	vitamin E, caffeic acid, iron, vitamin C	[42,101,102,103]
16.	*Allium schoenoprasum*; Chives; Purun-hnah	Leaves	Hypertension, Cancers, Diabetes, Asthma, Hyperlipidemia	allicin, S-allyl cysteine, quercetin, polyphenols, flavonoids	[42,104,105,106]
17.	*Brassica juncea*; Mustard; Tampui	Leaves, Stem/Decoction	Cancers, Obesity, Diabetes	sinapate, naringin, rutin, catechins, polyphenols	[42,107,108]
18.	*Cucurbita pepo* L.; Pumpkin; Maian	Leaves, seeds, pulp, plant, flowers/Paste, Cooked, Decoction, Oil	Hypertension, CVDs/Cataract	quercetin, glucosides, kaempferol, tocopherols, phenols	[42,109,110]
19.	*Sechium edule*; Chayote; Skut hnah	Leaves, Edible portion, roots, shoots/Extract	Cancers, Hypertension, Obesity, Diabetes, Hyperlipidemia, CVDs	gallic acid, chlorogenic acid, quercetin, rutin, flavonoids	[42,111,112,113]
20.	*Solanum khasianum*; Nightshade; Tawkte	Berries, Leaf, Root, stem, petiole/Extract	Cancers, Diabetes, CVDs, Hypertension	saponins, steroids, alkaloids, flavonoids, phenols	[42,114,115]

## Data Availability

The original contributions presented in this study are included in the article. Further inquiries can be directed to the corresponding author.

## Supplementary Materials

The following supporting information can be downloaded at: https://www.mdpi.com/article/10.3390/nu17203311/s1, Table S1. List of NTFPs, bioactive compounds/compound classes, and their potential usage; Table S2. List of LAFRs, bioactive compounds/compound classes, and their potential usage.

## Appendix A

Self-structured Questionnaire

### Appendix A.1. Sociodemographic Profile of the Respondents

(Please tick or fill in the appropriate option.)

1. Participant name ……………………
2. Age ……….
3. Gender ☐ Male ☐ Female ☐ Other
4. Tribe/Subtribe (please specify) …………….
5. Marital Status:

□ Single □ Married □ Divorced □ Widowed

6. Education Level:

□ No schooling □ Primary □ Secondary □ Higher secondary

□ Graduate □ Postgraduate

7. Occupation …………………
8. Monthly household income (Indian Rupees, INR)

□ Below ₹10,000

□ ₹10,001–₹30,000

□ ₹30,001–₹60,000

□ Above ₹60,000

9. Household Size ……………
10. Area of Residence ☐ Urban ☐ Peri-urban ☐ Rural

### Appendix A.2. Self-Structured 3-Day Dietary Recall Questionnaire

**Day**	**Time of Meal**	**Cereals and Millet**	**Pulses**	**Animal Foods**	**Green Leafy Vegetables**	**Other Vegetables**	**Roots and Tubers** **(Excluding Potato)**	**Fruits**	**Milk and Milk Products**	**Fats and Oils**	**Nuts and Seeds**
Day 1	Breakfast										
	Lunch										
	Dinner										
Day 2	Breakfast										
	Lunch										
	Dinner										
Day 3	Breakfast										
	Lunch										
	Dinner										
Mean Actual nutrient Intake											

### Appendix A.3. Top 5 Priority Food Preferences Questionnaire

The participants are asked to provide their top 5 priority food preferences under the appropriate column.

**Priority** **Options**	**Breakfast/** **Local Name**	**Lunch/** **Local Name**	**Dinner/** **Local Name**	**Snacks/** **Local Name**
1				
2				
3				
4				
5				

### Appendix A.4. Questions to Elicit Daily Food-Related Habits, Cooking Methods, Lifestyle Practices, and Any Traditional or Cultural Practices You Follow While Preparing or Consuming Food

Responses were noted by the interviewer for the following.

1. How do you consume/prepare animal foods?
2. Are there any ingredients or preparations unique to your culture or household?
3. How do you usually preserve food? Do you use fermentation, drying, or smoking?
4. What kinds of fats/oil do you use while cooking?
5. How often do you or your family consume processed foods?
6. Any food additives or flavoring agents added while cooking?
7. Are commercial products (such as baking soda or packaged foods) commonly used?
8. Do you or your community still follow foraging or gardening practices?
9. What cooking methods are common in your home? (steaming, boiling, grilling, etc.)
10. Are there any known or diagnosed NCDs or micronutrient deficiencies?
11. Has your cooking or eating habits changed over the years?
12. Do you skip meals? If so, why? Will it be replaced with any food?
13. How active are people in your community? Are smoking or alcohol habits, or tobacco, common?
14. Do you or your family participate in any physical exercise?
15. How often do you include homegrown or local fresh foods?
