# Supplementary material for: Assessing Dietary Patterns, Lifestyle Practices, and Forest Foods with Bioactive Potential to Address Micronutrient Deficiencies and Noncommunicable Diseases in Northeast India
Source: Nutrients. 2025 Oct 21;17(20):3311. doi: 10.3390/nu17203311 (PMC12567503; doi:10.3390/nu17203311)
Supplement: Supplementary file 1 [file nutrients-17-03311-s001.zip › nutrients-3890952-supplementary.pdf]

Supplementary material

Table S1. List of NTFPs, bioactive compounds/compound classes, and their potential usage.

S. No	Species Name. Local Name. Common Name	Parts used/ Preparation & Consumption Methods	Addressing the type of NCDs/micronutrient deficiency specific to the plant	Bioactive compounds/ Compound classes	References
1.	Callicarpa arborea Roxb Beauty berry Hnah kiah	Leaf, Bark/Decoction	Diabetes, Cancers (colon, Breast, Lung)	Clerodane diterpenoid, triterpenes, diterpenes, monoterpene, terpenoids, diterpenoids, triterpenoids, glycosides, saponins, steroids, phenols, tannins, flavonoids	**[42,43,44]**
2.	Catharanthus roseus (L.) Kumtluang Bright eyes	Leaf, whole plant, Root/Juice, decoction	Diabetes, Hypertension, Cardiovascular disease, Hodgkin's disease, lymphosarcoma, Wilkin's tumor, choriocarcinoma, neuroblastoma, carcinoma of the breast, and lungs	Vincristine, vinblastine, vindolidine, vindolicine, vindoline, vindogentianine, Catharoseumine, vindolinine, 14′,15′-didehydrocyclovinblastine, 17-deacetoxycyclovinblastine, 17-deacetoxyvinamidine, vinamidine, leurosine, catharine, cycloleurosine, leurosidine, and Ajmalicine	**[42,45,46]**
3.	Clerodendrum glandulosum Lindl. Phuihnam Hill glory bower	Leaf, stem, root/Decoction	Hypertension, Diabetes, Obesity, Heart disease	Caffeic acid, Verbacoside, and derivatives, scutellarin, luteolin, and apigenin	**[42,47]**
4.	*Helianthus annus;* Sunflower; Ni-hawi	Seeds, stem, roots, leaf, flower/Raw, Oil, Meal	Cancer (Colon), obesity, diabetes, Blood pressure, Coronary Heart Disease, Hypertension, Asthma/Bone health	Heliannone, apigenin, quercetin, kaempferol, luteolin, caffeic acid, caffeoylquinic acid, gallic acid, quinic acid, coumaric, protocatechuic, ferulic acid, sinapic acids, caffeic acid hexose I, p-Coumaric acid hexose, chlorogenic acid, 3,4-Di-O-caffeoylquinic acid, soquercitrin, 3,5-Di-O-caffeoylquinic acid,1,5-Di-O-caffeoylquinic acid, caffeic acid hexose II, and 4,5-Di-O-caffeoylquinic acid	**[42,48,49]**
5	*Oroxylum indicum;* Broken bones; Ar-chang-kawm	Bark/Decoction	Cardiac disorders, Cancer, Diabetes, Asthma	Baicalein, oroxylin A, chrysin, scutellarin, and ellagic acid	**[42,50]**
6	*Centella asiatica;* Indian Pennywort; Darbengbur/lambak	Leaf, Stem/Decoction, Powder	Diabetes, cancers	Quercetin, madecassoside, phytosterols, kaempferol, apigenin, rutin, asiaticoside, phenolic acids, catechin, flavonoids, madecassic acid, naringin, asiatic acid	**[42,51,52]**
7	*Artemisia vulgaris;* Mugwort; Sai	Plant/Decoction	Cancers (lung cancer, prostate cancer, myeloid leukemia, gastric cancer, and melanoma Diabetes, Hypertension, and Obesity	Artemisinin, artemisinin B, artemisic acid, apigenin, chrysoberyl, coumarin, diosmetin, eupafolin, eriodictyol, homoe-eriodictyol, isorhamnetin, jaceosidin, kaempferol derivatives, luteolin, morin, quercetin derivatives, quercetrin, rutin, tacrine, vitexin	**[42,53,54]**

**8**	*Zanthoxylum asiaticum;* Orange climber; Ching-it	Stem, bark, leaves, roots/Decoction	Cancers/Sickle cell anemia	Alkaloids, reducing sugars, phenolic compounds, tannins, flavonoids	**[42,55,56]**
**9**	*Mikania micrantha;* Bitter vine; Japan-hlo	Leaves, Whole plant/Decoction	Stroke, hypertension, diabetes, hypercholesterolemia, and cancers	Saponin, phenol, tannin, flavonoid, glycoside, cardiac glycoside, carotenoid, alkaloids, steroids, terpenoids	**[42,57,58]**
**10**	*Alstonia scholaris (L.) R.;* Devil's tree; Thuamriat	Bark, stem, leaves, and roots/Decoction	Cancers (Cervical, liver, epidermal, breast, leukemia), Diabetes, Heart-related disorders, Hypertension, Asthma	Isoquinoline, quinoline, bisindole, akuammicine, vallesamine, akuammiline, villalstonine, steroids, triterpenoids, phenols, flavonoid, alkaloid, kaempferol, catechin, ellagic acid, gallic acid	**[42,59,60]**
**11**	*Cannabis sativa L.;* Hemp; Trip Kanza	Seeds	Cancers (Colon, Prostate, and Lung), Heart disease, Diabetes, Hypertension/Bone health/	Limonene, caryophyllene, terpenoids, phytosterols, cannabinoids, polyphenols, phenolic compounds, lignans, stilbenes, flavonoids, resveratrol, quercetin, luteolin, isorhamnetin, naringenin, apigenin, catechins, caffeic acid, gallic acid, rosmarinic acid, ferulic acid, p-coumaric acid, syringic acid, 3,4-dihydroxybenzoic acid	**[42,61,62]**
**12**	*Magnolia champaca L;* Champak; Ngiau	Stem bark, Leaf/Miceration	Hypertension, Cancers	Trans-syringin, syringic acid, p-hydroxybenzoic acid, phenolic compounds, leucoanthocyanidins, hydroxypropenyl dimethoxyphenyl-β-D-glucoside, flavonoids, volatile oils, glycosides, digoxin, digitoxin, alkaloids, saponins	**[42,63,64]**
**13**	*Rubus ellipticus;* Yellow Himalayan raspberry; Zawngṭa	Leaves, fruits, shoots, root bark/Decoction, Juice	Cancer, diabetes	Rosamutin, pinfaensin, pyrogallol, β-sitosterol, quercetin 3-O-rhamnoside, ellagic acid pentoside, lambertianin C, ellagic acid rhamnoside, procyanidin dimer type B, cyanidin 3-O-rutinoside, cyanidin 3-O-glucoside, cyanidin 3-O-glucosyl-rutinoside, cyanidin 3-O-sophoroside, (+)-catechin, (-)-epigallocatechin gallate, aromadedrin, hyperoside, cis-tiliroside, kaempferol-3-O-hexoside, quercetin-3-O-glycoronide, kaempferol-3-O-β-D-glucuronic acid methyl ester, quercetin-3-O-β-D-glucopyranoside, tiliroside, astragalin, imperatorin, rubusin A&B, esculetin, esculin, casuariin, casuarinin, casuaricitin, pedunculagin, caffeic acid glucoside, sinapaldehyde, syringaldehyde, pinobanksin, hesperidin, quercetin, rutin, naringenin, galangin, apigenin, delphinidin, cyanin, kaempferol, trans-cinnamic acid, vanillic acid, 4-hydroxybenzoic acid, 3-hydroxybenzoic acid, m-coumaric acid, chlorogenic acid, caffeic acid, gallic acid, ellagic acid.	**[65,66]**
**14**	Termitomyces heimii; Wild mushrooms; Pasawntlung	Edible portion/Cooked	Hyperlipedemia, Cancers	Quercetin, protease, kaempferol, saponins, serine, cerebrosides, ergostanes, fatty acid amides, tannins, epicatechins, catechins, rutin, isoquercitrin, quercitrin, chlorogenic acid, caffeic acid, gallic acid, ellagic acid, stilbenes, lignans, flavonoids, phenolic acids	**[67,68]**
**15**	Cinnamomum tamala; Indian bay leaf; Theipui	Leaves	Hypercholesterolemia, cancer (Colon), cardiac diseases, diabetes	α-humulene, α-muurolene, nerolidol, spathulenol, cinnamyl acetate, hydrocinnamyl acetate, cinnamic acid, coumarin, benzaldehyde, salicylaldehyde, acetophenone, 3-phenyl propanal, pinocarvone, borneol, bornyl acetate, geraniol, linalool, carophyllene oxide, caryophyllene, germacerene D,	**[69-71]**

				curcumenol, curzerenone, furanodiene, furanodienone, furanogermenone, sesquiterpenoids, monoterpenoid hydrocarbons, oxygenated monoterpenes, sesquiterpene hydrocarbons, oxygenated sesquiterpenes, phenylpropanoids, terpenoids, triterpenoids, steroids, phenolic acid, flavonoids, tannins, alkaloids, glycosides, anthroquinones, anthocyanins, coumarins, cinnamaldehyde, trans-cinnamaldehyde, eugenol, methyl eugenol, eugenol acetate, kaempferol-3-O-glycopyranoside, quercetin-3-O-rutinoside, quercetin-3-O-sophoroside, kaempferol-3,7-di-O-rhamnopyranoside, α-pinene, myrcene, camphene, p-cymene, limonene, γ-terpinene, terpinen-4-ol, p-cymen-8-ol, α-terpineol	

Table S2. List of LAFRs, bioactive compounds/compound classes, and their potential usage.

S. No	Species Name. Local Name: Common Name	Parts used/ Preparation & Consumption Methods	Addressing the type of NCDs/micronutrient deficiency specific to the plant	Bioactive compounds/ Compound classes	References
**1.**	Beta Vulgaris L. Beet root Beetroot	Rhizome/Raw, Juice	Colorectal, Prostate, Breast cancer/anemia	Lutein, β-carotene, lycopene, Apigenin, 8-O-xyloside, Isoscutellarein 7-O-glucoside, Caffeoyl-6-(3,4-dihydroxy benzoyl) β-D-glucoside	**[42,72,73]**
**2**	Carica papaya L. Thingfanghma Papaya	Seed, Fruit, Leaf, Sap/Paste, Raw	Prostate cancer, liver cancer, breast Cancer, Diabetes, Obesity, Cardiovascular diseases, and Asthma	Quercetin, caffeic acid, p-coumaric acid, gallic acid, ferulic acid, lycopene, alpha-pinene, and limonene	**[72,74]**
**3**	Citrus maxima Sertawk Pomelo	Whole plant, whole fruit, albedo, Peel, Leaf, Pulp, and Seed/Raw	Hypertension, Colon cancer, Obesity, Diabetes, Pancreatic cancer, liver cancer, cardiovascular disease, and Asthma/Osteoporosis	Naringin, Hesperidin, naringenin, limonene, 5-demethyl nobiletin, diosmin, hesperetin, nobiletin, quercetin, Limonin, nomilin, limonexic acid, fortunellon, rutin, tangeretin, auraptene, and neohesperidin	**[72,75-78]**
**4**	Citrus limon (L.) Nimbu Lemon	Fruit, Stem, Leaf, Peel/Juice, Decoction	Neurodegenerative diseases, cardiovascular disease, Hypertension, diabetes, cancer/Scurvy, and osteoporosis	Polyphenols, terpenes, limonene, flavonoids, vitamin C, citric acid, malic acid, flavonoids such as naringin, eriocitrin, hesperidin, narirutin, rhoifolin, vitexin, diosmin rioctitrin, hesperidin, diaosmin, carotenoids, terpineol, fellander, camhenium, citrain, and coumarin	**[72,79,80]**
**5**	Colocasia esculenta (L.) Dawl Elephant ear	Stem, shoots, flowers, sap, bulbs, leaves/juice, Boiled, stewed	Cancer, Diabetes/Anemia	Terpenoids, glycosides, alkaloids, flavonoids such as isovitexin 3′-O-glucoside, isovitexin, leteolin-7-O-glucoside, vitexin X′′-O-gluco-side, vicenin-2, isoorientin, orientin-7-O-glucoside, orientin	**[42,72,81]**

				tryptophan, vitexin, luteolin-7-O-rutinoside, luteolin-7-O-glucoside, 1-O-feruloyl-D-glucoside, 1-O-caffeoyl-D-glucoside, 1-O-feruloyl-D-glucoside, and rosmarinic acid , tannins, polyphenols such as 6,2',4'-trimethoxyflavanone, luteolin-6,8-Cdiglucoside, gallic acid, benzoic acid, chlorogenic acid, quercetin, astilbin, catechin, and kampferol, with rutin, vitexin, caffeic acid, ellagic acid, saponins, mono galactosyl diacyl glycerols (MGDGs) and digalactosyl diacyl glycerols	
**6.**	Crassocephalum crepidioides (Benth.) S. Moore Buar thau Fireweed ragleaf	Leaf/Paste	Diabetes, Cancer, Obesity, Hypertension/Anemia	β-cubebene, α-farnesene, gallic acid, catechin, rutin, aquercetin, chlorogenic acid, caffeic acid, ellagic acid, hexadecenoic methyl ester, Isochlorogenic acid, quercetin, gallic, chlorogenic, coumarins, kaempferol glycosides, benzofuran, benzofuranone, linolenic acid, α-caryophyllene	**[42,72,82,83,84]**
**7.**	Cucurbita maxima Duchesne Mai Pumpkin	Fruit, flesh, seed, leaf, and peel/raw, boiled, steamed	Diabetes, obesity, cancer (prostate, breast, gastric, lung, and colorectal cancers), Hypertension, cardiovascular disease/anemia	Gallic acid, Protocatechuic acid, Vanillic acid, chlorogenic acid, caffeic acid, coumaric acid, ferulic acid, Kaempferol, Isoflavones, quercetin, α-tocopherol, isoquercetin, Cucurbitacin, terpenoids, triterpenoids, and γ-tocopherol.	**[42,72,85,86,87]**
**8.**	Curcuma longa L. Aieng Turmeric	Rhizome/Powder, Juice	Cancer (breast, colon, pancreatic, prostate, lung cancer), Diabetes, Cardiovascular Disease/Anemia	curcumin, Ar-turmerone, β-turmerone, α-zingiberene, ar-curcumene, β-sesquifelandreno, curcumin II, curcumin III, dimethoxy-, bisdemethoxy-curcumin	**[42,72,88]**
**9.**	Cucumis sativus L. Fanghma Cucumber	Leaf, flesh, seeds, Skin/Raw, decoction	Hypertension, Diabetes, Prostate and colon cancers	Cucurbitacin B, -6-nonenol, E-2-nonenal, E,Z-2,6-nonadienal, E-2-nonenal,Z-3-nonenol, 3-nonenal, pentadecanal, 9,12,15-octadecatrienal and 9,17-octadecadienal	**[42,72,90,91]**
**10.**	Dillenia pentagyna Roxb Kaihzawl Elephant Apple	Leaf, Bark, Fruits/Decoction	Cancer, Diabetes, Heart health	Botulin, Botulinic acid, lupeol, Morolic acid, Rhamnetin 3-O-glucoside, Dill hydro quercetin 5-glucoside, Naringenin 7-galactosyl (1-4) glucoside and Naringenin-4'-O-[4-O-(β-D-glucopyranosyl)]-β-D-xylopyranoside, β-sitosterol, gallic acid, syringic acid, synaptic acid, and diploic acid	**[42,72,92]**
**11.**	*Zingiber officinale;* Ginger; Sawhthing	Rhizome/Raw, Decoction	Diabetes, Cardiovascular disease, Cancer (Colon, breast Prostate cancers)/Anemia, Osteoporosis	gingerol, shogaol, zingerone, paradol, Diarylheptanoids, 4-gingesulfonic acid, 6-gingesulfonic acid, and shogasulfonic acids A, B, C and D, β-sitosterol, daucosterol, stigmast-4-en-3,6-dione, 6β-hydroxystigmast-4-en-3-one, stigmast4-en-3-one,6-gingerol, 10-gingerol, 6-shogaol and 10-shogaol,8-gingerol, 8-shogaol, 8- paradol, 10 – paradol, 6- paradol, quercetin, rutin, epicatechin, and stigmasterol	**[42,72,93,94]**

**12.**	*Phyllanthus emblica L;* Indian Gooseberry; Sunhlu	Fruit/Raw, Juice	Diabetes, Cancer, Asthma/Anemia, Scurvy	Ellagic acid, pyrogallol, corilagin, gallic acid, chebulagic acid, flavonoid, Vitamin C (ascorbic acid), quercetin, chebulic acid, chebulanin acid, caffeic acid, citric acid, epicatechin	**[42,72,95,96]**
**13.**	*Hylocereus undatus; Dragon fruit; Dragon fruit*	Flesh, seeds, and peels	Obesity, Diabetes, cancers (breast, colon), and cardiovascular disease/Anemia	Quercetin, chlorogenic acid, phloridzin, catechin, sinapic acid, gallic acid, rutin, caffeic acid, vanillic acid, syringic acid, hesperidin, p-coumaric acid, salicylic acid, protocatechuic acid, p-hydroxybenzoic acid, ascorbic acid (Vitamin C), thiamin (Vitamin B1), niacin, riboflavin, β-carotene, lycopene, betacyanin, tocopherol (Vitamin E), Iron	**[42,97,98,99]**
**14.**	*Ananas comosus L;* Pine apple; Lakhuih	Pulp, Leaf, Root, Skin, core, stem/Raw, Juice, Paste	Heart disease, diabetes, and cancers/Bone & Oral health	Bromelain, phytosterols, ferulic acid, flavonoids, vitamin C, epicatechin, phenolic compounds, organic acids, catechin, gallic acid	**[42,100,101,102]**
**15.**	*Phyllostachys edulis;* Bamboo shoot; Mautuai	Edible portion/Boiled	Coronary heart disease, Cancer, diabetes, obesity/Micronutrient deficiency	β-sitosterol, stigmasterol, vitamin E, campesterol, ferulic acid, protein, iron, catechin, stigmastanol, caffeic acid, protocatechuic acid, p-coumaric acid, p-hydroxybenzoic acid, chlorogenic acid, ergosterol, vitamin C, cholesterol, syringic acid, minerals	**[42,103,104,105]**
**16**	*Allium schoenoprasum;* Chives; Purun-hnah	Leaves	Hypertension, Cancer, Diabetes, Asthma, Hyperlipidemia	S-allyl cysteine, S-methyl cysteine, S-methyl cysteine sulfoxide (SMCS), diallyl sulfide, diallyl sulphide, allicin, allyl trisulfide, polysulfanes, quercetin, polyphenols, tannins, flavonoids, gallic acid, ferulic acid, cinnamic acid, N-caffeoyltyramine, carotenoids, anthocyanins, alkaloids, saponins	**(42,106,107,108)**
**17**	*Brassica juncea;* Mustard; Tampui	Leaves, Stem/Decoction	Cancers (Lung, Colon, Breast, Prostate), obesity, Diabetes	1-O-β-D-glucopyranosyl sinapate, naringin, rutin, epicatechin gallate, epigallocatechin gallate, catechin, proanthocyanidins, protocatechuic acid, p-hydroxybenzoic acid, p-coumaric acid, ferulic acid, hydroxy-ferulic acid, vanillic acid, caffeic acid, gallic acid, sinapic acid, sinapine, sinapoyl glucose, vanillin, p-hydroxybenzaldehyde, chlorogenic acid, sugar alcohols, amine, oxoproline, hydroxylamine, polyphenols	**[42,109,110]**
**18**	*Cucurbita pepo L.;* Pumpkin; Maian	Leaves, seeds, pulp, plant, flowers/Paste, Cooked, Decoction, Oil	Hypertension, Heart health/Cataract	Quercetin 3-galactoside, kaempferol 3-glucoside, isorhamnetin 3-rutinoside, rutin, myricetin, astragalin, isoquercetin, kaempferol, quercetin, α-tocopherol, γ-tocopherol, sinapic acid, ferulic acid, p-coumaric acid, caffeic acid, chlorogenic acid, gallic acid, protocatechuic acid, 4-hydroxybenzoic acid, vanillic acid	**[42,111,112]**
**19**	*Sechium edule;* Chayote; Skut hnah	Leaves, Edible portion, roots, shoots/Extract	Cancers, Hypertension, Obesity, Diabetes, Hyperlipidemic, Heart disease	Gallic acid, chlorogenic acid, caffeic acid, syringic acid, vanillic acid, p-hydroxybenzoic acid, ferulic acid, p-coumaric acid, naringenin, quercetin, myricetin, rutin, phlorizin, phloretin, galangin, apigenin, hesperetin, glycinein	**[42,113,114,115]**
**20**	*Solanum khasianum;* Nightshade; Tawkte	Berries, Leaf, Root, stem, petiole/Extract	Cancers, Diabetes, Cardiovascular diseases, Hypertension	Geranylgeraniol, vitamin E, Spirost-8-en-11-one, 3-hydroxy-(3β,5α,14β,20β,22β,25R)-, cis-Vaccenic acid, 9,12-Octadecadienoic acid (Z,Z)-, Tetradecanoic acid, Myristoleic acid, 2-Pyrrolidinone, 1-methyl, 4H-Pyran-4-one, 2,3-dihydro-3,5-dihydroxy-6-methyl-, Phthalic acid, isobutyl nonyl ester, saponins, steroids,	**[42,116,117]**

	triterpenoids, alkaloids, flavonoids, glycosides, phenols, tannins	
